# Rush Medical College, Chicago

**Published:** 1844-02

**Authors:** 


### RUSH MEDICAL COLLEGE, CHICAGO.

The Mississippi Valley is remarkable for many things, but will soon be as much so for its medical colleges as for aught else. These multiply astonishingly. They grow as rapidly as Jonah's gourd. One lies down at night to rest from a hard day's toil, and after a deep sleep, such as fell upon Adam, he wakes, and lo! by his side is a medical college, "complete from turret to foundation-stone." They come not singly merely, but by twos and threes—in populous cities, and amid "populous solitudes of bees and birds" as well. Not quicker or more sudden did Roderic Dhu's archers, at blast of bugle-horn, start up from rock and tree, than do these institutions appear at trumpet or even penny-whistle call of some aspiring grandson of Apollo. With one flourish of the serpent-bound staff of him worshipped

> In Argolis, beside the echoing sea, they come, thick-clustering over the land,

> As when the potent rod
> Of Amram's son, in Egypt's evil day
> Wav'd round the coast, up-call'd a pitchy cloud
> Of locusts.

We have now a complete chain of them, enough to make a veritable *cordon sanitaire*. Disease is hemmed in—is "cabined, cribbed, confined"—and can have neither egress nor ingress without challenge from trusty sentinel. Tracing them on the map, they make a figure resembling an ill-shaped battledoor. Beginning with that at New Orleans, where, as stated in another paragraph, a new college building is just completed, we go to St. Louis and find two more; next to Jacksonville where there is a fourth; then to Chicago and count a fifth; turning thence down the lake shore, we find a sixth at Laporte; proceeding to the southern shore of Lake Erie, Cleveland gives us a seventh, and Willoughby, eighteen miles distant, an eighth; crossing the country and following the Ohio, Cincinnati gives the ninth; a *detour* to Lexington and we have a tenth; Louisville makes the eleventh; another *detour* to Nashville, completes the dozen; and if we count one that is in a formative stage at Madison, Indiana, we have a clean baker's dozen. Seven States, containing 13 medical colleges, some of which are almost within call of each other!

The youngest of these, is the *Rush Medical College*, at Chicago—a city which 12 years ago was a fur-trading establishment, and now contains some thousands of inhabitants. The charter of the college dates from March, 1837, but no organization took place until the past autumn. In October the present faculty was appointed; at a subsequent period, means were provided to defray the expenses of a chemical apparatus and dispensary; and the first session of the college opened on the 4th December last. The following gentlemen compose the faculty: Daniel Brainard, M.D., Professor of Anatomy and Surgery; James V. Z. Blaney, M.D., Professor of Chemistry and Materia Medica; John M'Lean, M.D., Professor of Theory and Practice of Medicine; and M. L. Knapp, M. D., Professor of Obstetrics, and Diseases of Women and children. Dr. Brainard lately filled with great credit the chair of Anatomy in the University of St. Louis. Dr. Blaney, though young, is full of talent, and has that enthusiasm in his profession so necessary to a teacher, and which cannot fail of success. Of the other gentlemen we know nothing, but doubt not they are every way worthy their two colleagues.

Of Dr. Brainard's excellent address, delivered at the opening of the college, we shall say nothing now. We have laid it aside with a number of others, which, at some favorable moment, we intend to take up and *eviscerate* for the edification and instruction of our readers. The best wish that we can give him and the Rush Medical College (and we give it heartily) is, that the spirit of the great man whose name it bears, may rest and remain with them. C.

